# Low-Dose Catheter-Directed Thrombolysis for Massive Pulmonary Embolism: A Case Report Highlighting Dosing Considerations in Asian Patients

**DOI:** 10.1155/cric/3210422

**Published:** 2025-11-29

**Authors:** Zhongjian Tang, Yang Song, Min Zhang, Mengyan Li, Jingjing Shang, Aiya Shu, Kunkun Wang, Yun(Lucy) Lu

**Affiliations:** ^1^Department of Intensive Care Unit, Fuling Center Hospital of Chongqing, Chongqing, China; ^2^Department of Pharmacy, St. Joseph Medical Center, Tacoma, Washington, USA; ^3^Department of Pharmacy Services, Boston Medical Center, Boston, Massachusetts, USA; ^4^Department of Pharmacy, Brigham and Women's Hospital, Boston, Massachusetts, USA; ^5^FMH Pharmacy, Fairbanks Memorial Hospital, Fairbanks, Alaska, USA; ^6^Department of Pharmacy, Hennepin Healthcare System, Minneapolis, Minnesota, USA

**Keywords:** embolism, race, thrombolysis, thrombosis

## Abstract

Clinical management of massive pulmonary embolism is challenging when active hemorrhage, a contraindication to thrombolytics, is concurrently present. We describe a successful attempt in using low-dose catheter-directed thrombolysis (CDT) in a high-risk patient with absolute contraindications to systemic thrombolysis. A 69-year-old Asian female with cardiac arrest was brought to a resource-limited rural hospital. The patient underwent 50 min of cardiopulmonary resuscitation (CPR) before regaining the pulse but remained in cardiogenic shock. Computerized tomography (CT) of the chest found massive PE. The patient was found with multiple fractures and subarachnoid hemorrhage. Catheter-directed embolectomy was performed without clinical improvement. A low-dose CDT with alteplase was attempted by giving 5 mg over 2 h with a repeated session 24 h later for a total of 10 mg. The patient started improving, was extubated on Day 9, and transferred out of the ICU on Day 15. Low-dose CDT in massive PE could be lifesaving despite the presence of absolute alteplase contraindications. Patients with contraindications, a high risk of bleeding, or of Asian race may benefit more from the low-dose alteplase regimen.

**Summary**

Low-dose CDT with alteplase is a potential approach for the safe and effective management of massive or high-risk PE, despite the presence of major hemorrhage complications.

## 1. Introduction

Massive or high-risk pulmonary embolism (PE) has an in-hospital mortality rate exceeding 50% [1]. Thrombolytics can be lifesaving and remain the gold standard for massive or high-risk PE. However, major hemorrhage and fatal or intracranial bleeding are significantly more frequent in patients receiving systemic thrombolytics [2]. Retrospective evidence and single-arm studies suggest that, for patients with contraindications to intravenous thrombolytics, catheter-directed thrombolysis (CDT) offers an alternative treatment option, particularly in those with high bleeding risk, providing rapid improvement and low rates of bleeding complications [1]. However, evidence derived from large randomized controlled trials is still lacking regarding whether CDT affects patients' prognosis compared to systemic thrombolytics. We report a case of low-dose catheter-directed alteplase use in a high-risk PE Asian patient with absolute contraindications to systemic thrombolysis.

## 2. Case Report

A 69-year-old Asian female (60 kg), with onset of chest pain 8 h prior, became pulseless 30 min after presentation to a resource-limited rural hospital. She underwent cardiopulmonary resuscitation (CPR) for 50 min before regaining her pulse and subsequently was admitted for cardiogenic shock and acute hypoxemic respiratory failure. The patient's past medical history includes idiopathic pericardial effusion.

Upon examination, the patient was afebrile, with a blood pressure (BP) of 84/59 mmHg, a heart rate (HR) of 79 bpm, a respiratory rate of 25, altered mental status with a Glasgow Coma Scale (GCS) of 6, a fraction of inspired oxygen (FiO_2_) of 80%, and an oxygen saturation (SpO_2_) of 77% on mechanical ventilation. Blood gas results indicated respiratory acidosis. D-dimer elevated to 3.56 mg/L with increased prothrombin time (PT) and partial thromboplastin time (aPTT). Acute kidney injury was suspected with a serum creatinine of 1.40 mg/dL. Cardiac troponin I and N-terminal-pro B-type natriuretic peptide (NT-proBNP) were elevated at 0.88 ng/mL and 4620 pg/mL, respectively. ST segment depression in lead aVL and T wave inversion in leads AVF, V1–V4 were shown in the electrocardiogram (EKG).

Bedside ultrasound showed moderate tricuspid regurgitation, left ventricular diastolic dysfunction, a small amount of fluid in the hepatorenal recess, and no signs of lower extremity venous thrombosis. A computerized tomography (CT) of the chest found a thrombus in the main pulmonary artery and other pulmonary arteries in the left upper and lower lobes, consistent with acute massive PE. The patient also suffered from pulmonary contusion, rib, and sternal fractures with hemothorax and intra-abdominal bleeding, likely as a result of CPR.

Twenty-four hours after CPR, a 6 French vascular sheath was placed through the femoral vein to guide a 4 French pigtail catheter into the pulmonary artery. The distal tip fragmented the thrombus and suctioned out a rice-size thrombus. However, the patient remained hypotensive and hypoxic after the embolectomy. After informed consent was obtained from the family, alteplase 5 mg was given to the patient via CDT. Three hours after local thrombolysis, BP dropped from 108/68 to 86/53 mmHg, hemoglobin (Hgb) dropped from 107 to 86 g/L, and pleural effusion and ascites increased mildly. A transfusion of red blood cells (RBCs) 600 mL improved Hgb to 96 g/L and BP to 105/65 mmHg. Day 1 post-CDT, the patient had an PaO_2_/FiO_2_ ratio of 68; a systolic BP goal of 90 mmHg or mean arterial pressure (MAP) > 65 mmHg was maintained by norepinephrine at 1.5 *μ*g/kg/min.

**Figure 1:**
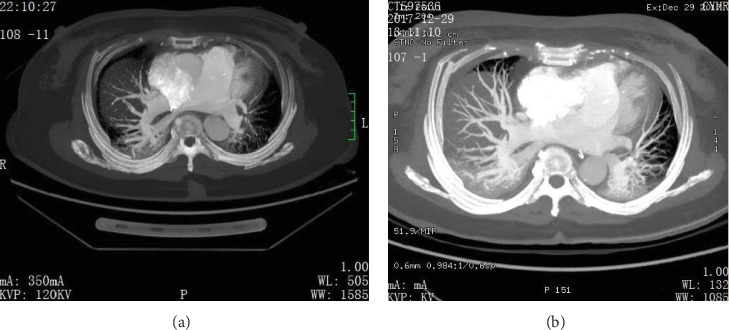
(a) CTPA before CDT versus (b) CTPA post-CDT.

The CDT with alteplase 5 mg was repeated 24 h after the first session. Another 600 mL RBC was transfused to maintain Hgb at 99 g/L. Twelve hours after the second 5 mg dose, the PaO_2_/FiO_2_ ratio was improved to 108, norepinephrine requirement decreased to 0.16 *μ*g/kg/min, central venous pressure (CVP) went from 11 cm H_2_O to 7 cm H_2_O, lactic acid dropped to 1.3 mmol/L, 24-h urine output increased to 3950 mL, and NT-proBNP dropped to 1730 ng/mL. Once the coagulation profile normalized, low-dose heparin infusion was initiated with the aPTT goal of 60–80 s. Computerized tomography pulmonary angiogram (CTPA) was repeated on Day 4 (Figure 1), showing the recanalization of pulmonary arteries.

On Day 6, the patient regained responses to speech with a GCS of 9. The weaning of ventilation started on Day 9. The patient was extubated on Day 11 and transferred out of the ICU on Day 15.

## 3. Discussion

Both the CHEST and the European guidelines recommend alteplase 100 mg IV over 2 h for a massive or high-risk PE unless there is a contraindication [2, 3]. Systemic thrombolysis carries an estimated 20% risk of major hemorrhage including a 3%–5% risk of hemorrhagic stroke for the treatment of PE. The alternative options for patients with contraindications to systemic thrombolysis include catheter interventions with or without thrombolysis or surgical pulmonary embolectomy [3, 4]. In this report, the patient underwent CDT after failing mechanical embolectomy in the presence of multiple contraindications: recent subarachnoid hemorrhage, hemothorax, intra-abdominal bleeding, and recent intracranial hemorrhage. CDT allows direct administration of thrombolytics into the pulmonary arteries using a lower thrombolytic dose to decrease thrombus burden immediately.

Although various dosing strategies have been reported, the optimal dose of alteplase in CDT is unknown. The SEATTLE II study was a prospective multicentral trial evaluating the safety and effectiveness of ultrasound-assisted catheter-directed thrombolysis (UACT) low-dose alteplase 24 mg over 24 h for the treatment of PE. However, this is a single-arm study with moderate to severe bleeding events occurring with 10% of patients [5]. In addition, although this study is racially diverse, no Asian patient is included [5]. The OPTALYSE PE trial investigated the optimum duration and dose of tPA with the acoustic pulse thrombolysis procedure for submassive PE without reporting race [6]. The dose of 10 mg alteplase in this case was about 50% of the dose reported by the above EU/US studies. It has been suggested that low-dose alteplase (0.6 mg/kg) intravenously in Asian patients with acute ischemic stroke did not show inferiority to the standard dose (0.9 mg/kg) [7]. This low-dose strategy for alteplase in ischemic stroke is approved in Japan and adopted by many Asian hospitals. Thus, the individualized lower dosing strategy is selected in this patient case with the consideration of racial differences in responding to alteplase. Additionally, the administration time was shortened to 2 h to induce faster onset with improved efficacy. Although this case suggests CDT with low-dose alteplase was effective in decreasing the proximal thrombus burden and reducing right ventricular (RV) strain without major complications, the available clinical evidence is not sufficient to devise a hospital treatment protocol in areas with resource-limited and high medication acquisition cost. UACT has emerged as a promising approach in the management of massive PE since FDA approval in 2014. Studies have found that a fixed low-dose UACT of alteplase 10–20 mg over 15 h rapidly improved hemodynamic and RV dysfunction with low bleeding rates in patients with intermediate and high-risk PE [6]. UACT was hypothesized to provide superior clinical efficacy compared to standard CDT by using high frequency, low-power ultrasound waves to improve thrombus penetration and safety profile in patients with high bleeding risks [7]. However, a retrospective study showed similar improvement in pulmonary artery systolic pressure (PSAP), the right-to-left ventricle (RV/LV) ratio, and intensive care unit hospital length of stay when comparing UACT and CDT. A trend toward a lower bleeding rate was observed in the CDT group (8.3% vs. 12.9%, *p* = 0.74) [8]. Ultrasound assistance was not available at the time of CDT in this patient case. Further prospective studies are warranted to compare clinical outcomes and complications of low dosing strategy between USAT and CDT delivery modalities in high-risk PE.

There is a paucity of data regarding the optimal timing of CDT in high-risk PE. The average time from presentation to UACT was 25.4 h from a 91-patient retrospective study with three high-risk PE patients. The study found a significant reduction in pulmonary arterial pressure and 8% of patients suffered from bleeding complications [9]. Another retrospective study showed that intermediate-risk patients may benefit from early USAT (< 24 h) rather than delayed initiation (> 24 h) with significant improvement in pulmonary arterial pressures and cardiac index; however, the RV/LV ratio change was not statistically significant [10]. While the timing from the onset of symptoms to the first dose of CDT was about 32 h in this patient, further studies are required to confirm these findings and establish the appropriate timeline for initiation of CDT with or without ultrasound.

There is an increasing use of CDT for high bleeding risk PE in the United States and Europe over the past decade [11]. Compared to surgical embolectomy or systemic thrombolysis, CDT is associated with fewer complications and a shorter in-hospital stay, which may lead to reduced costs [12]. Randomized controlled trial evidence, such as the HI-PEITHO study, which compares CDT with anticoagulation alone for acute intermediate high-risk PE and is expected to complete in 2026, is urgently needed to provide high-quality data on clinical outcomes [13].

## 4. Conclusion

Low-dose CDT with alteplase was a potential approach for safe and effective management of massive or high-risk PE despite the presence of major hemorrhage complications. Future studies are warranted to guide dosing strategies and compare clinical outcomes and complications of low dosing strategies between USAT and CDT delivery modalities in resource-limited settings.

## Data Availability

The data that support the findings of this study are available on request from the corresponding author. The data are not publicly available due to privacy or ethical restrictions.
